# Species composition and invasion risks of alien ornamental freshwater fishes from pet stores in Klang Valley, Malaysia

**DOI:** 10.1038/s41598-020-74168-9

**Published:** 2020-10-14

**Authors:** Abdulwakil Olawale Saba, Ahmad Ismail, Syaizwan Zahmir Zulkifli, Muhammad Rasul Abdullah Halim, Noor Azrizal Abdul Wahid, Mohammad Noor Azmai Amal

**Affiliations:** 1 Department of Biology, Faculty of Science, Universiti Putra Malaysia, 43400 UPM Serdang, Selangor, Malaysia; 2 School of Agriculture, Lagos State University, Epe Campus, Epe, 106101 Lagos Nigeria; 3 School of Biological Sciences, Universiti Sains Malaysia, 11800 Gelugor, Penang, Malaysia; 4 Institute of Advanced Studies, University of Malaya, 50603 Kuala Lumpur, Malaysia

**Keywords:** Conservation biology, Freshwater ecology, Invasive species

## Abstract

The ornamental fish trade has been considered as one of the most important routes of invasive alien fish introduction into native freshwater ecosystems. Therefore, the species composition and invasion risks of fish species from 60 freshwater fish pet stores in Klang Valley, Malaysia were studied. A checklist of taxa belonging to 18 orders, 53 families, and 251 species of alien fishes was documented. Fish Invasiveness Screening Test (FIST) showed that seven (30.43%), eight (34.78%) and eight (34.78%) species were considered to be high, medium and low invasion risks, respectively. After the calibration of the Fish Invasiveness Screening Kit (FISK) v2 using the Receiver Operating Characteristics, a threshold value of 17 for distinguishing between invasive and non-invasive fishes was identified. As a result, nine species (39.13%) were of high invasion risk. In this study, we found that non-native fishes dominated (85.66%) the freshwater ornamental trade in Klang Valley, while FISK is a more robust tool in assessing the risk of invasion, and for the most part, its outcome was commensurate with FIST. This study, for the first time, revealed the number of high-risk ornamental fish species that give an awareness of possible future invasion if unmonitored in Klang Valley, Malaysia.

## Introduction

As a global hobby, fishkeeping is cherished by both young and old people. Besides, it contributes to the improvement of human well-being by building responsibility in children, managing stress in adults, and helping the elderly to cope with their critical physical and psychological states^1^. However, some hobbyists lack information or are careless regarding the preservation of natural biodiversity^2^. When aquarium fishes attain large unmanageable sizes or become very aggressive, they are likely to be dumped into local waters^3^. Hobbyists may also get tired of certain fishes or get uncomfortable with their excessive breeding habits^4^. This route of introduction is probably the least regulated in many countries^5^. As a result, many fish species have deliberately or inadvertently been introduced into the native ecosystems especially in areas where the aquarium fish industry is very active. Therefore, the rate of introduction and spread of alien fish species has been eased by human activities and the impacts are on environmental and/or socioeconomic^1^.

In Malaysia, aquarium trade stands as the second most important route through which alien fish species are introduced into inland water bodies, after aquaculture^6^. From 2007 to 2014, the trade bloomed from the value of USD 18.9 million to US 85.0 million in the country, indicating the importance of the industry^7,8^. However, a larger proportion of the ornamental fishes in the aquarium business are alien, thereby increasing the probability of dumping, and risk of invasion^6^. In terms of propagule pressure, the aquarium route may out-perform aquaculture as a pathway of species invasion if the dumping of unwanted aquarium fishes continues to increase with minimal or no regulation and control^9^. Moreover, in countries like Japan, Singapore, Taiwan, and Australia, dumping of ornamental fishes has been the main route of species introduction^2^.

Risk evaluation is essential as a first step in evaluating the hazard of alien species to the native ecosystem and species biodiversity^10^. Consequently, different risk assessment methods have been developed and used to understand and predict the invasiveness of alien fish species around the world including the Fish Invasiveness Screening Test (FIST)^2,11^, Fish Invasiveness Screening Kit (FISK)^12–15^, and Aquatic Species Invasiveness Screening Kit (AS-ISK)^16,17^. Paradoxically, there is no single study on the invasion risk of alien ornamental freshwater fishes in Malaysia using any of these tools. Since prevention has been identified as the backbone of alien invasive species management, it is necessary to identify and understand the existing or potential risks of various routes of aquatic species invasion^18^.

Being the most developed and populated region in the country, this research aims not only to identify the ornamental fishes in pet stores, but also to ascertain the invasion risks of top aliens present in stores within Klang Valley, Malaysia. This area is well known to harbour many ornamental fish pet stores coupled with its sport fishing activities. Besides, highly populated areas are more prone to fish introduction^19^. The area is also home to several local freshwater bodies including rivers, lakes, and streams that are susceptible to the dumping of aquarium fishes. Moreover, this study will not only provide baseline information for future research, but also serve as a tool for a more focused and intensified effort towards the management of alien fish species, and in the long run, help prevent adverse impacts of fish species invasion in the local ecosystems.

## Methods

### Ethics statement

The fishes were sampled, handled, and sacrificed according to the methods approved by Institutional Animal Care and Use Committee, Universiti Putra Malaysia. All methods were carried out in accordance with relevant guidelines and regulations.

### Study area

Klang Valley is situated at the centre of the west coast of Peninsular Malaysia and covers five major areas such as the Federal Territory of Kuala Lumpur, Gombak, Hulu Langat, Klang and Petaling, where they cover an approximate area of 2,832 km^2^. The region cuts across cities such as Ampang Jaya, Kuala Lumpur, Klang, Petaling Jaya, Shah Alam, and Subang Jaya^20^. This area was selected as a result of being urbanized and with a heavy population of over 4 million people which represents about 16% of the entire country’s population^21^. To see the spread and frequency of the various species in pet stores around the area, only retail stores were included in the study. Likewise, the retail stores are more widely distributed and probably serve as the major route through which alien fish species may be introduced into nearby local water bodies. The study took place throughout the year 2019 and essentially surveyed all identified and accessible ornamental fish pet stores within Klang Valley, Malaysia.

### Sampling procedures

Since there was no official sampling frame to determine the total number of stores within the sampling area, this study relied on the data from Department of Fisheries, Malaysia, and Google maps to collate and locate all available pet stores in the area. The stores were screened for possible inclusion or exclusion from the survey, after each visit. Pet stores that had too few freshwater fishes, usually just for display purposes and those that deal only with marine fishes were also excluded from the survey. In each pet store, all available fish species were observed and documented by listing the common and scientific names. Species that could not be identified immediately were purchased and taken to the laboratory for further identification.

### Species identification and checklists

The fishes were identified using a combination of keys and publications from previous studies^22–28^. Information on the order, family, species, conservation status, native distribution, climatic zone, and the threat to humans were ascertained using FishBase^29^ and Eschmeyer's Catalog of Fishes^30^. Each species was designated as alien or native based on information gathered. However, identification challenges were experienced with a few fish samples and so were not included for further analysis to avoid misidentifications and erroneous conclusions. Eventually, checklists of all recorded alien and native fishes were presented.

### Descriptive analyses

Descriptive analyses were carried out using Microsoft Excel (Office 365, Version 2016, Microsoft Corp., Berkshire, UK) to present the charts and graphs showing occurrence frequencies and other descriptive information about the recorded native and alien fish species. These include information regarding families, orders, geographical origin, conservation status, native distribution, climatic zone, and the threat to humans. To facilitate the analysis, all recorded fish species, after identification, were listed and each was coded for each aquarium shop as 0 if the fish species was unavailable, and 1 for available fish species. Varieties of any particular species were taken as a single species to avoid taxonomic confusion.

### Percentage of occurrence, conservation status, climatic zone and the threat to human

Recorded alien fish species were distinguished and classified based on the percentage of occurrence (popularity) as low (≤ 25%), moderate (26—50%), and high (≥ 51—100%). Also, following previous study^31^ and using information from FishBase^29^, the recorded information was detailed as follows: native range (continents and sub-continents), basic climatic zones (temperate, tropical, sub-tropical), conservation status (such as not assessed (NA), critically endangered (CR), near threatened (NT), least concern (LC), vulnerable (VU), data deficient (DD))^32^ and potential threats to humans such as harmless (H), toxic (T), venomous (V), traumatogenic (TR) and poisonous (P)^29^.

### Comparison of fish occurrence by origin

The Mann Whitney *U* test was used to compare the occurrence of native versus alien fishes in the stores, while Spearman rank correlation was also carried out to identify the strength, direction, and significance of the relationship between the number of alien fish species recorded and the total number of species recorded in the pet stores using IBM SPSS Statistics for Windows, Version 23.0. Armonk, NY, USA. These were done after exploring the data and confirming that they did not satisfy the conditions for parametric statistical analyses.

### Risk assessment

Risk assessment to quantify the invasiveness of the alien fish species was carried out. In the first stage, the criteria for selection of recorded fish species which were included in the assessment are; percentage of occurrence greater than 25% in the sampled pet stores/existing history of establishment, percentage of occurrence less than 25% in the stores/ previous reports of the species presence in Malaysian water bodies, and percentage of occurrence less than 25% in the stores/evidence that the species has been established in many other countries.

Secondly, the invasion risk was explored in two phases. First, using the Fish Invasiveness Screening Test (FIST)^2,11^ with minor modification. Thereafter, the Fish Invasiveness Screening Kit (FISK) v2 was applied^33^. FIST serves as a rapid tool that presents an index for screening latent biological characteristics associated with invasion. For this study, characteristics considered include maximum adult length, propagule pressure (expressed as % of occurrence frequency of a species in surveyed pet stores), history of establishment, breeding in the wild, competition with native species, diet plasticity, temperature tolerance, and tolerance to low dissolved oxygen.

After ascertaining the characteristics of a species from consulted sources, the traits were classified as absent or low ( +), moderate (+ +), and high (+ + +) for each species based on the eight considered features^2,11,25,34–43^. For computation, only a criterion where a species qualifies for ‘ +  +  + ’ is quantified using the ‘number 1′, while a score below that such as ‘ +  + ’ or ‘ + ’ is assigned a score of ‘0′ since they are not considered in the final risk analysis^2,11^. The scoring criteria for FIST are shown in Table 1.

**Table 1:** Scoring criteria for Fish Invasiveness Screening Test.

No	Traits	Criteria	Score
1	Maximum adult length	20.0 to 40.0 cm	+
		40.1 to 60.0 cm	+ +
		> 60.0 cm	+ + +
2	Relative frequency	0.0 to 10.0%	+
		10.1 to 30.0%	+ +
		30.1 to 100.0%	+ + +
3	History of establishment	0 to 7 countries	+
		8 to 15 countries	+ +
		> 15 countries	+ + +
4	Ability to breed in the wild	Low	+
		Moderate	+ +
		High	+ + +
5	Competition with native species	Low/Absent	+
		Moderate	+ +
		High	+ + +
6	Feeding plasticity	Specialist/not voracious	+
		Opportunist/moderately voracious	+ +
		Generalist/voracious	+ + +
7	Temperature tolerance	Low	+
		Moderate	+ +
		High	+ + +
8	Tolerance to low dissolved oxygen	Low	+
		Moderate	+ +
		High	+ + +

The percentage value of the Frequency Distribution (FD%) for screened criteria was enumerated for each species after which invasiveness was measured from the calculated FD% value based on risk level. When a species is with an FD% value for ‘ +  +  + ’ of above 50% for all screened criteria, they were classified as high-risk, while species with values between 30 and 50% were classified as moderate-risk and those below 30% as low-risk.

Using FISK, species were analysed to double-check and ascertain the level of risk from FIST. The FISK is a tool that evaluates invasion risk of fish species based on a set of 49 questions that fall into two groups such as ‘biogeography/history’ and ‘biology/ecology’, each having three and five subcategories, respectively^4,33^. Since every response in FISK for each species has a certainty score (1 = very uncertain; 2 = mostly uncertain; 3 = mostly certain; 4 = very certain) for each species, a ‘certainty factor’ (CF) was computed as ∑(CQi)/(4 × 49) (i = 1, …, 49), where CQi is the certainty for the question i, 4 is the maximum achievable value for certainty (i.e. ‘very certain’) and 49 is the total number of questions in the FISK tool. The CF, therefore, ranges from a minimum of 0.25 for all 49 questions with certainty score equal to 1 to a maximum of 1 for all 49 questions with certainty score equal to 4. In addition to the published literature consulted, databases such as the Database of Introduced Aquatic Species (DIAS) (https://www.fao.org/fishery/dias/en), Global Invasive Species Database (GISD) (https://www.iucngisd.org/gisd/), Centre for Agriculture and Bioscience International (CABI) (https://www.cabi.org/), FishBase (https://www.fishbase.org) and the United States Fish and Wildlife Service (USFWS) (www.usfws.gov) provided information were used to complete the analysis.

The power of FISK v2 to correctly discriminate between non-invasive (low and medium-risk) and invasive (high-risk) species gives a measure of its predictive potential^44^. Therefore, FISK was calibrated for Peninsular Malaysia using the ROC (Receiver Operating Characteristics) after generating reports of 23 species^15,44^. To achieve this, a priori species categorization based on perceived invasiveness (i.e. invasive or non-invasive) was carried out using information available from the Invasive Species Specialist Group database (ISSG) (http://www.iucngisd.org/gisd/) and FishBase^12,29,44–46^. ISSG provides information on the establishment status of fishes in non-native regions and gives general information on their ecology, distribution, impact, and management. For the hybrid fish, a priori risk ranking was based on the taxon with the highest invasiveness risk^44^.

The ROC analysis presents a curve which is a graph of sensitivity versus 1- specificity (sensitivity versus specificity). Here, sensitivity refers to the proportion of invasive and non-invasive fish species, respectively, that are accurately recognized by the FISK tool. The area under the curve (AUC) helps to determine the precision of the calibration analysis. An AUC of 1.0 means that the test is completely accurate and both sensitivity and specificity are equal to 1.0 with no false positives (i.e. non-invasive species categorised as invasive) or false negatives (i.e. invasive species categorised as non-invasive). Contrarily, an AUC of 0.5 means that the test is 100% inaccurate and is unable to separate true positives (i.e. actual invasive species) from true negatives (i.e. actual non-invasive species)^14,44^.

Assessments were carried out by two assessors (fish ecologists) leading to individual and universal ROC curves. The universal ROC curve was computed using the mean scores from both assessors. From the global ROC curve, the best threshold (cut-off) value that maximises the true positive rate (true invasive categorized as invasive) and minimises the false-positive rate (true non-invasive categorized as invasive) was ascertained using Youden’s *J* statistic^47^ and the closest point to the top-left part of the plot with perfect sensitivity or specificity^45,48,49^. A ‘delta’ value was computed to determine the difference between scores obtained by both assessors^44^. Also, the CFs for the 23 species were compared for the two assessors using the Mann Whitney *U* test^15^. All statistical analysis was performed using IBM SPSS Statistics for Windows, Version 23.0. Armonk, NY, USA.

## Results

**Figure 1:**
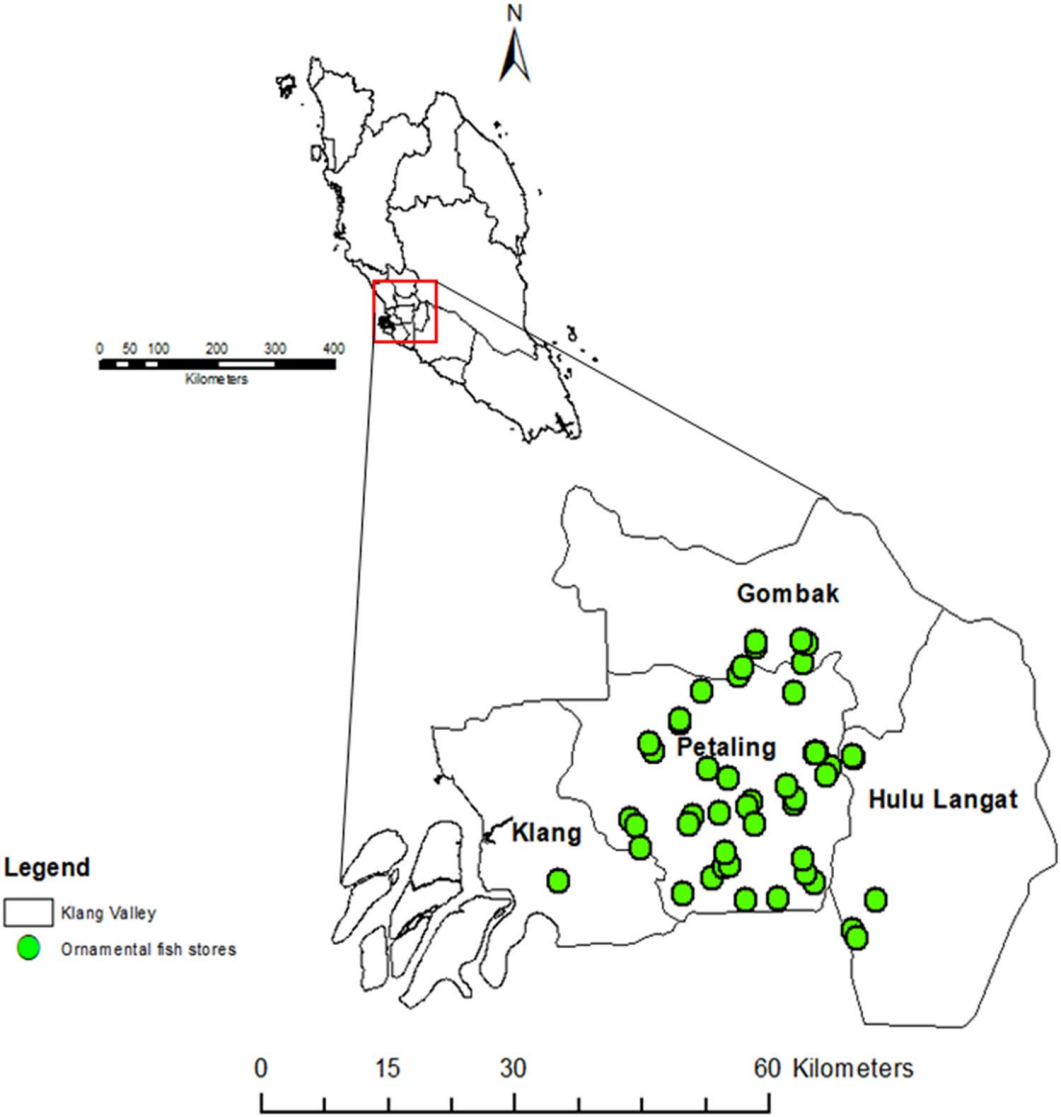
The administrative districts of the surveyed ornamental fish stores within Klang Valley, Malaysia. To generate this figure, the shapefile containing administrative zones of Malaysia was sourced from DIVA-GIS (http://www.diva-gis.org/gdata) and imported into ArcGIS Desktop version 10.2 (https://desktop.arcgis.com/en/arcmap).

A total of 60 ornamental fish stores located within the five districts that cut across Klang Valley area were successfully surveyed (Fig. 1).

### Species checklist, orders, and families

Checklists of all recorded alien (85.66%, n = 251) and native (14.33%, n = 42) fish species are presented in Appendix 1 and Appendix 2, respectively. Species with no official common names were considered as not available (NA) and the list was arranged based on families. The alien fishes belong to 18 orders including Gymnotiformes, Acanthuriformes, Acipenseriformes, Anabantiformes, Centrarchiformes, Cichliformes, Lepisosteiformes, Myliobatiformes, Osteoglossiformes, Atheriniformes, Gobiiformes, Tetraodontiformes, Cyprinodontiformes, Polypteriformes, Cypriniformes, Characiformes, Siluriformes, and Perciformes. Most of them belong to the order Cichliformes (28.2%, n = 71) followed by Siluriformes (18.7%, n = 47) and Characiformes (15.9%, n = 40), while Perciformes, Gymnotiformes, Gobiiformes and Centrarchiformes recorded the least number of species (0.40%, n = 1), respectively.

**Figure 2:**
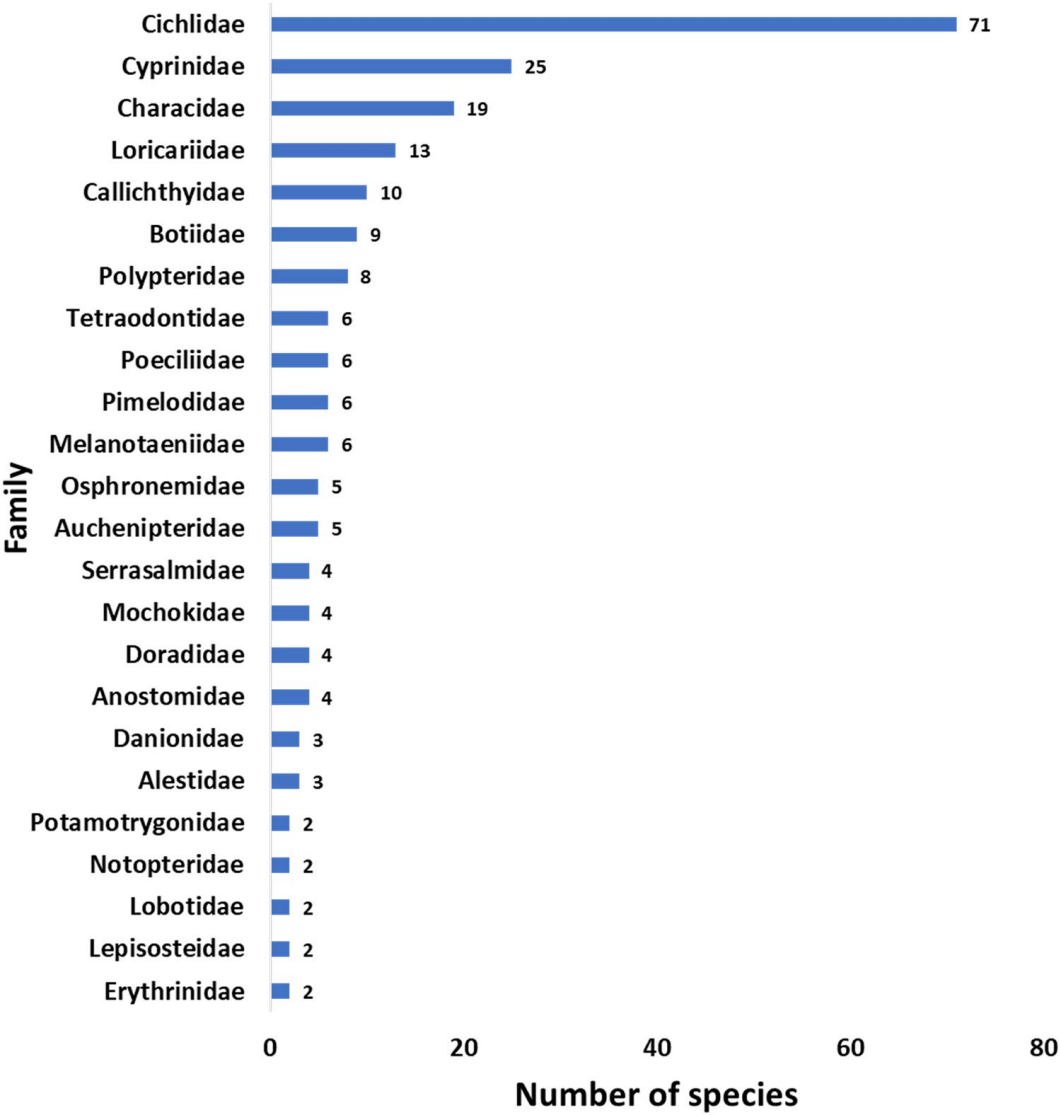
Alien fish families encountered in ornamental fish pet shops within Klang Valley with number of species in each family.

Out of the total of 53 families recorded, Cichlidae (n = 71) which belongs to the order Cichliformes and occupied 100% of all Cichliformes recorded, followed by family Cyprinidae (n = 25) which occupied about 67.6% of all Cypriniformes (Fig. 2). A total of 29 families had only one species each and these include Acipenseridae, Anabantidae, Apteronotidae, Arapaimidae, Ariidae, Bryconidae, Centrarchidae, Chalceidae, Channidae, Clariidae, Claroteidae, Cobitidae, Ctenoluciidae, Cynodontidae, Distichodontidae, Gasteropelecidae, Gastromyzontidae, Gobiidae, Gyrinocheilidae, Hepsetidae, Monodactylidae, Mormyridae, Pangasiidae, Polyodontidae, Prochilodontidae, Procatopodidae, Siluridae, and Tanichthyidae.

### Percentage of occurrence and natural distribution

Based on percentage of occurrence (popularity) at the 60 pet stores, only 4% (n = 11) of the alien fishes had high occurrence, 8% (n = 19) occurred moderately, while the remaining 89% (n = 221) had low occurrence. The top occurring species with percentage occurrence > 25% included two-hybrid cichlids that had popularity (occurrence percentage) greater than 50% with goldfish (*Carassius auratus*) having the highest popularity of 83.3% occurrence, while silver dollar (*Metynnis hypsauchen*) had the lowest with the popularity of 26.7% occurrence (Appendix 1). The largest percentage of alien fish species recorded in fish pet stores originated from South America (44%), followed by Asia (25%) and Africa (20%).

### Climatic zone, conservation status and the threat to human

**Figure 3:**
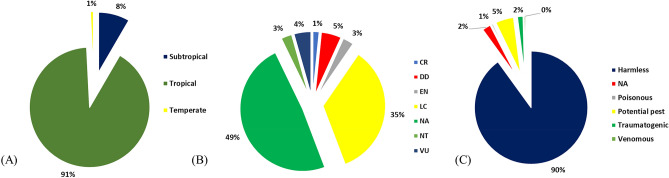
(**A**) Climatic zone of alien fish species; (**B**) IUCN Red List conservation status of recorded alien fish species where NE = not evaluated, CR = critically endangered, NT= near threatened, LC = least concern, VU = vulnerable, DD = data deficient, and EN = endangered fish species; (**C**) Recorded alien fish species and their level of threat to humans; NA = not applicable. This figure was generated using Microsoft Excel (Office 365, Version 2016, Microsoft Corp., Berkshire, UK).

A total of 91% (n = 228) of the recorded fish species belong to the tropical climatic zone, while 8% (n = 21) and 1% (n = 2) belong to the subtropical and temperate regions, respectively. Based on the IUCN Red List conservation status, 3% (n = 7) each of the recorded species are endangered (EN) and near threatened (NT), while 4% (n = 11) and 1% (n = 4) are vulnerable (VU) and critically endangered (CR), respectively. Menarambo cichlid (*Paretroplus menarambo*), maingano (*Pseudotropheus cyaneorhabdos*)*,* giant barb (*Catlocarpio siamensis*) and Siamese tiger perch (*Datnioides pulcher*) were designated as critically endangered, while shovel nose tiger catfish (*Pseudoplatystoma magdaleniatum*)*,* zebra loach (*Botia striata*)*,* putitor masheer (*Tor putitora*)*,* lipstick goby (*Sicyopus jonklaasi*)*,* Boseman’s rainbowfish (*Melanotaenia boesemani*)*,* Denison's barb (*Sahyadria denisonii*) and striped catfish (*Pangasianodon hypophthalmus*) are endangered, while elongate mbuna (*Chindongo elongatus*)*,* emperor cichlid (*Aulonocara nyassae*)*,* silver carp (*Hypophthalmichthys molitrix*)*,* panda cory (*Corydoras panda*)*,* Blyth's loach (*Syncrossus berdmorei*) and royal knifefish (*Chitala blanci*) are near threatened. Interestingly, 5% (n = 13) of all alien fishes were recorded as potential pests including species such as north African catfish (*Clarias gariepinus*), vermiculated sailfin catfish (*Pterygoplichthys disjunctivus*)*,* goldfish (*Carassius auratus*)*,* common carp (*Cyprinus carpio*) among others^29^. Only 2% of the species (n = 4) are traumatogenic^29^ (Fig. 3).

### Species comparison in stores by origin

Non-native fish species were significantly (*U* = 159.0, p < 0.001) higher in number (*Mdn* = 25) compared to the native fish species (*Mdn* = 6). Besides, Spearman rank correlation indicated a significant positive association between the number of non-native fish and the total number of fish species (r = 0.969, p < 0.001) recorded in the pet stores.

### Invasiveness screening using FIST and FISK

Based on the selection criteria, the top 23 alien species including 16 of the most occurring (> 25%) species and seven other species that satisfy the selection criteria were subjected to invasiveness screening. FIST showed that seven (30.43%), eight (34.78%) and eight (34.78%) species were considered to be of high, medium and low invasion risks, respectively. The high-risk species include north African catfish (*Clarias gariepinus*), goldfish (*Carassius auratus*), common carp (*Cyprinus carpio*), guppy (*Poecilia reticulata*), flowerhorn (cichlid hybrid), redtail catfish (*Phractocephalus hemioliopterus*) and suckermouth catfish (*Hypostomus plecostomus*) (Table 2)*.*

**Table 2:** Outcome of Fish Invasiveness Screening Test for selected top alien fishes from ornamental pet stores within Klang Valley, Malaysia.

Species assessed	Invasiveness screening criteria								Invasion risk (%FD)
	1	2	3	4	5	6	7	8	
*Poecilia latipinna*	+	+ + +	+ +	+	+ +	+	+	+	Low (12.50)
*Gymnocorymbus ternetzi*	+	+ + +	+	+	+	+	+	+	Low (12.50)
*Danio rerio*	+	+ + +	+	+	+	+	+	+	Low (12.50)
*Betta splendens*	+	+ + +	+	+	+	+	+	+	Low (12.50)
*Trichogaster lalius*	+	+ + +	+	+	+	+	+	+	Low (12.50)
*Pethia conchonius*	+	+ + +	+	+	+	+ +	+	+	Low (12.50)
*Barbonymus gonionotus*	+ +	+ + +	+	+ + +	+	+	+	+ +	Low (25.00)
*Piaractus brachypomus*	+ + +	+	+	+	+ + +	+ +	+	+ +	Low (25.00)
*Pangasianodon hypophthalmus*	+ + +	+ + +	+	+	+	+ + +	+	+ +	Medium (37.50)
*Pterygoplichthys pardalis*	+ +	+	+ +	+ + +	+ + +	+ +	+	+ + +	Medium (37.50)
*Pygocentrus nattereri*	+ +	+	+ +	+ +	+ + +	+ + +	+	+ + +	Medium (37.50)
*Puntigrus tetrazona*	+	+ + +	+	+ +	+	+ +	+	+ + +	Medium (37.50)
*Astronotus ocellatus*	+ +	+ + +	+	+ + +	+ + +	+ +	+	+ + +	Medium (50.00)
*Pterygoplichthys disjunctivus*	+ + +	+	+ +	+ + +	+ + +	+ +	+	+ + +	Medium (50.00)
*Arapaima gigas*	+ + +	+	+	+ + +	+ + +	+ +	+ +	+ + +	Medium (50.00)
*Cichla ocellaris*	+ + +	+	+ +	+ + +	+ + +	+ +	+ + +	+ +	Medium (50.00)
*Poecilia reticulata*	+	+ + +	+ + +	+ + +	+ + +	+ +	+ +	+ + +	High (62.50)
Cichlid hybrid (flowerhorn)	+ + +	+ + +	+	+ + +	+ + +	+ +	+ +	+ + +	High (62.50)
*Phractocephalus hemioliopterus*	+ + +	+ + +	+	+	+ + +	+ + +	+ +	+ + +	High (62.50)
*Hypostomus plecostomus*	+ + +	+ + +	+ + +	+ + +	+ +	+ +	+ +	+ + +	High (62.50)
*Cyprinus carpio*	+ + +	+ + +	+ + +	+ + +	+ + +	+ +	+ +	+ + +	High (75.00)
*Carassius auratus*	+ + +	+ + +	+ + +	+ + +	+ + +	+ +	+ + +	+ + +	High (87.50)
*Clarias gariepinus*	+ + +	+ +	+ + +	+ + +	+ + +	+ + +	+ + +	+ + +	High (87.50)

% FD = frequency distribution (%) for (+ + +) score, low-risk =  < 30%, moderate-risk = 30 to 50%, high-risk =  > 50.1%

1: Maximum adult length (cm), 2: Occurrence frequency, 3: History of establishment, 4: Breeding in the wild, 5: Competition with native species, 6: Diet plasticity, 7: Temperature tolerance, 8: Low dissolved oxygen tolerance.

**Figure 4:**
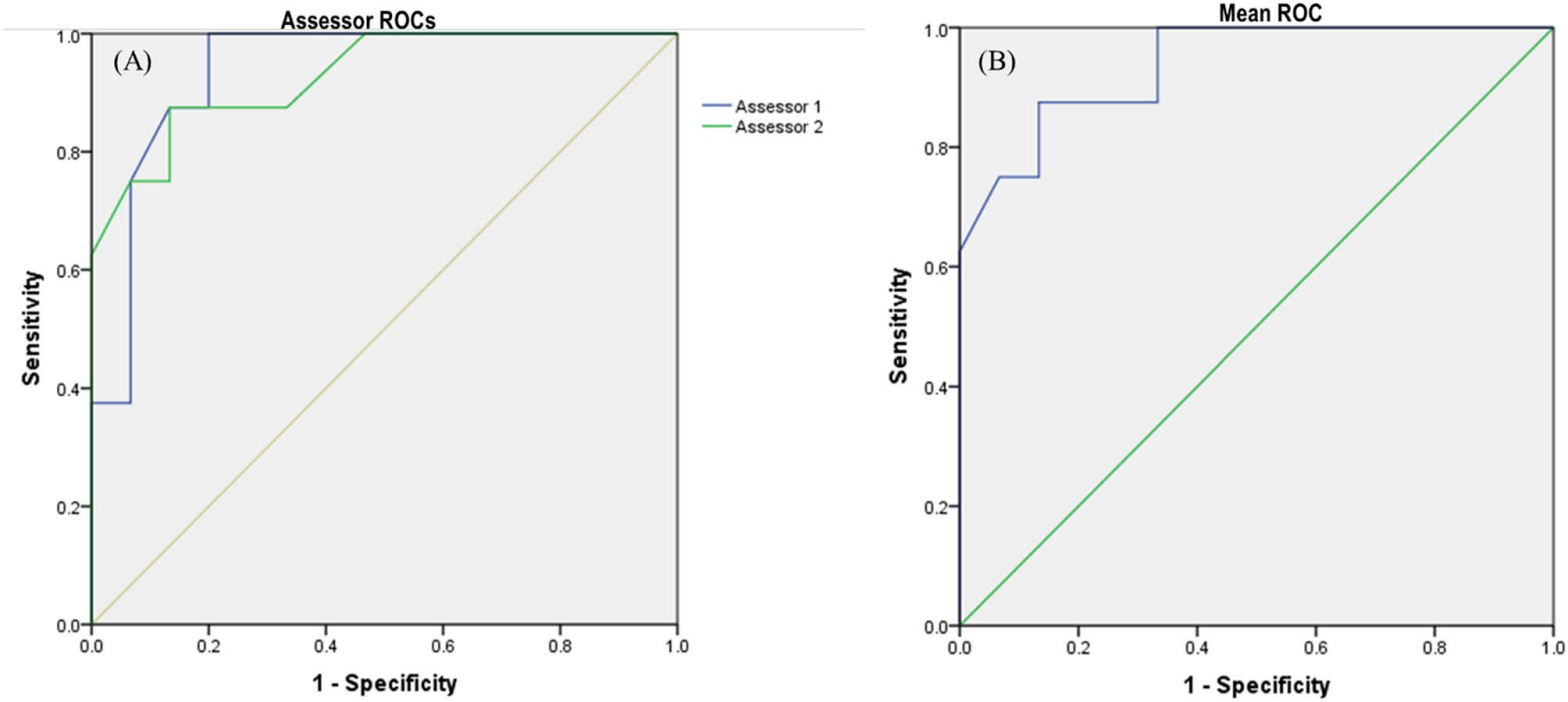
(**A**) Individual receiver operating characteristics (ROC) curves. (**B**) Mean receiver operating characteristics curves for two assessors on 23 freshwater aquarium fish species in Klang Valley, Malaysia using FISK.

To calibrate FISK, AUCs (0.938, 1.000–0.840 CI for Assessor 1 and 0.929, 1.000–0.819 CI for Assessor 2) from the ROCs were close for both assessors. As a result, an overall ROC was computed using the mean FISK v2 scores and this gave an AUC value of 0.938, 1.00–0.839 (Fig. 4). This indicates the ability of FISK to discriminate between invasive and non-invasive species based a priori classification. The best threshold as provided by Youden’s *J* was chosen to be 17, which was the score used to calibrate FISK to distinguish between medium and high-risk species. Because this threshold resulted in no invasive species being ranked as low risk, we retained the FISK v2 score interval from -15 to 0 for low-risk species.

The obtained mean FISK scores resulted in 14 (60.9%) species classified as medium risk and 9 (39.1%) species as high risk. The a priori classification gave 8 (34.8%) species as invasive and 15 (65.2%) species as non-invasive. Out of the 15 species classified a priori as non-invasive, FISK was able to correctly classify 93.3% as medium-risk and out of the 8 species, a priori classified as invasive FISK assessment was able to classify 87.5% as high-risk. Furthermore, FISK scores for the 15 species a priori classified as non-invasive ranged from 6.5 to 24, while the 8 species a priori classified as invasive ranged from 14 to 32.5. Individual assessor scores ranged from 5 for arapaima (*Arapaima gigas*) to 36 for north African catfish (*Clarias gariepinus*), while the mean assessor scores ranged from 6.5 for redtail catfish (*Phractocephalus hemioliopterus*) to 32.5 for north African catfish (*Clarias gariepinus*) (Table 3). Five species including redtail catfish (*Phractocephalus hemioliopterus*), arapaima (*Arapaima gigas*)*,* red piranha (*Pygocentrus nattereri*)*,* black tetra (*Gymnocorymbus ternetzi*) and flowerhorn (cichlid hybrid) had the lowest FISK scores, while peacock bass (*Cichla ocellaris*), goldfish (*Carassius auratus*), amazon sailfin catfish (*Pterygoplichthys pardalis*), common carp (*Cyprinus carpio*) and north African catfish (*Clarias gariepinus*) had the highest FISK scores in ascending order.

**Table 3:** Fish species assessed with FISK v2 for ornamental fish species within Klang Valley, Malaysia. High-risk species were selected based on a threshold value of ≥ 17.

Species name	Common name	FISK score	CF	Invasiveness
		Mean ± SE	Mean ± SE	
*Clarias gariepinus*	North African Catfish	32.5 ± 3.5	0.825 ± 0.015	High
*Cyprinus carpio*	Carp	32.0 ± 2.0	0.825 ± 0.035	High
*Carassius auratus*	Goldfish	26.0 ± 2.0	0.825 ± 0.025	High
*Pterygoplichthys pardalis*	Amazon sailfin catfish	26.0 ± 3.0	0.905 ± 0.025	High
*Cichla ocellaris*	Peacock bass	25.0 ± 2.0	0.935 ± 0.025	High
*Hypostomus plecostomus*	Suckermouth catfish	24.0 ± 1.0	0.830 ± 0.020	High
*Pterygoplichthys disjunctivus*	Vermiculated sailfin catfish	24.0 ± 1.0	0.935 ± 0.015	High
*Astronotus ocellatus*	Oscar	21.0 ± 1.0	0.910 ± 0.020	High
*Poecilia reticulata*	Guppy	19.0 ± 1.0	0.850 ± 0.030	High
*Pangasianodon hypophthalmus*	Striped catfish	15.0 ± 1.0	0.855 ± 0.015	Medium
*Barbonymus gonionotus*	Silver barb	15.0 ± 2.0	0.880 ± 0.000	Medium
*Piaractus brachypomus*	Pirapitinga	14.5 ± 0.5	0.880 ± 0.010	Medium
*Poecilia latipinna*	Molly	14.0 ± 1.0	0.890 ± 0.010	Medium
*Betta splendens*	Siamese fighting fish	13.5 ± 0.5	0.845 ± 0.025	Medium
*Danio rerio*	Zebrafish	13.0 ± 1.0	0.880 ± 0.010	Medium
*Pethia conchonius*	Rosy barb	11.5 ± 1.5	0.865 ± 0.015	Medium
*Trichogaster lalius*	Moonling gourami	10.0 ± 1.0	0.840 ± 0.040	Medium
*Puntigrus tetrazona*	Sumatra barb	8.5 ± 2.5	0.890 ± 0.010	Medium
*Pygocentrus nattereri*	Red piranha	8.0 ± 2.0	0.875 ± 0.035	Medium
*Gymnocorymbus ternetzi*	Black tetra	8.0 ± 1.0	0.860 ± 0.020	Medium
Cichlid hybrid	Flowerhorn	8.0 ± 1.0	0.830 ± 0.010	Medium
*Arapaima gigas*	Arapaima	7.0 ± 2.0	0.935 ± 0.015	Medium
*Phractocephalus hemioliopterus*	Redtail catfish	6.5 ± 1.5	0.795 ± 0.005	Medium

**Figure 5:**
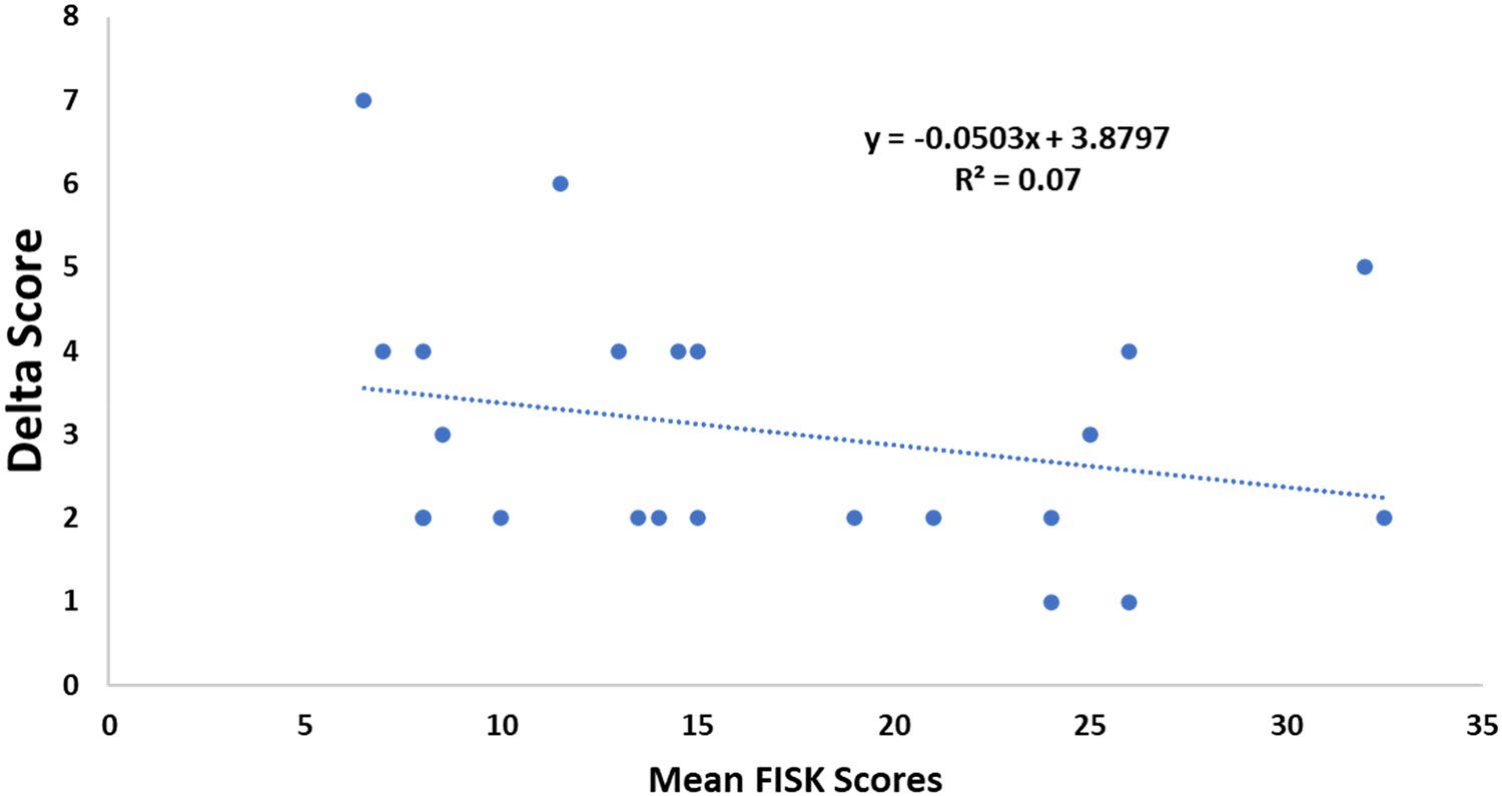
Relationship between mean FISK v2 scores and the assessor difference (delta values).

Delta scores ranged from 1 to 7 and there was a significant negative relationship (p < 0.05) with mean FISK scores (Fig. 5). The average CF for all responses (mean ± SE) was 0.87 ± 0.008.

## Discussion

A vast majority of the alien species recorded in this study originate from the tropics, in line with the scenario reported in Greece, Brazil, Canada, and USA^1,4,31,50,51^. This trend signals that many species from that region are of desirable ornamental characteristics possessing the ability to survive and thrive at least in a controlled environment. Furthermore, Malaysia falls within the tropical region, and the high proportion of tropical alien aquarium fishes recorded in this study signals the possibility of establishment since the environment may be very suitable. Moreover, over a half (51%) and a third (35%) of the cichlid species originate from South America and Africa, which are the first and third in terms of origin for species reported in this study, respectively. South America has the most diverse fish fauna on earth which currently numbers over 9000 of freshwater and marine species combined, about 27% of all fishes around the world^52^. This huge number of species is likely to consist of a correspondingly high number of freshwater ornamental fish species. This possibly explains the reason why most of the species encountered originate from South America.

Furthermore, family Cichlidae probably consists of species with more desirable ornamental characteristics compared to family Cyprinidae. This finding is in agreement with a previous study^31^. However, it is slightly different from the report of Magalhães and Jacobi^51^ who reported Cyprinidae as the most popular, followed by Poeciliidae. Besides, all of the alien cyprinid fishes recorded in pet stores originate from other Asian countries. This gives an idea of the abundance of cyprinids in the region and the risk of further introduction of new species. The proximity of Malaysia to other Asian countries could also facilitate this situation since importation from these countries would likely be easier compared to countries in farther continents.

Occurrence frequency may likely predict the magnitude of release (propagule pressure) of alien fish into native ecosystems^3^. For example, Duggan et al.^9^ in their study of ornamental fish species available in Canada and the USA established a relationship between the occurrence frequency of alien fish families in stores and their subsequent introduction and establishment. Goldfish (*Carassius auratus*), freshwater angelfish (*Pterophyllum scalare*) and Sumatra barb (*Puntigrus tetrazona*) were the most occurring alien fishes in this study. This, in part, is similar to the findings in Pacific Northwest that cuts across USA and Canada^1^, and for San Francisco Bay-Delta region in California, USA^4,53^. A previous report with an even higher frequency of occurrence is probably due to the broader coverage of that study^3^.

This study also indicates that alien fish species contributed the most to the total number of species available for sale. Although chain stores were not encountered during the survey, previous studies have noted that independent retail stores generally sold a higher number of different fish species compared to chain stores^54^.

Alien fish species have been recorded from diverse ecosystems in Malaysia, and these include, but not limited to drainage areas, mining pools, lakes, reservoirs, swamps, streams, and rivers^6,55–57^. The presence of some of these species including peacock bass (*Cichla ocellaris*), striped catfish (*Pangasianodon hypophthlamus*), piranha (*Serrasalmus* sp.), arapaima (*Arapaima gigas*), north African catfish (*Clarias gariepinus*) and tilapia (*Oreochromis* spp.) has been attributed to purposeful introduction into local waters^6,58^. Besides, some of them are well adapted to the environment, grow rapidly, feed on local fishes, and some are considered invasive^59,60^. For example, the Amazon sailfin catfish (*Pterygoplichthys pardalis*) has established a self-breeding population in Langat River, Selangor, and more than 10 tonnes of the fish were reportedly been caught from the Skudai River, Johor. They may have outcompeted the native fish species owing to their hardy characteristics^61,62^. This species also disrupt the environment since they dig the river bottom to make burrows to lay eggs. As a result, they tend to contribute to erosion and sedimentation of the river bottom^63^.

As one of the top-ten countries that produce ornamental fishes of freshwater origin, the ornamental fish industry in Malaysia is a sizeable one and includes the collection, breeding, and marketing which creates jobs for the locals and generates foreign exchange for the country^64–66^. However, there are pieces of evidence that the ornamental fish trade is a vital route through which alien fish gets introduced and translocated^4,50,67,68^. In fact, due to their behaviour and potential to attain large sizes, alien species belonging to the genera *Hypostomus* and *Pterygoplichthys* likely get released especially when they become too large for most domestic aquaria^3^.

Just like in this study, FIST was earlier used for selected ornamental fish species in Brazil^2^ and India^11^. However, a slight modification of their methods warranted the inclusion of occurrence frequency of alien fish species as part of the criteria for risk analysis. This may be the reason why some known high-risk species turned out to be of moderate-risk due to their low to moderate occurrence in the stores, which reduced their scores during analysis. Some of the high to moderate-risk species are known to have established breeding populations in Malaysian inland waters. For example, the peacock bass (*Cichla ocellaris*), vermiculated sailfin catfish (*Pterygoplichthys disjunctivus*), Amazon sailfin catfish (*Pterygoplichthys pardalis*), arapaima (*Arapaima gigas*), alligator gar (*Atractosteus spatula*) and pirapitinga (*Piaractus brachypomus*) have been encountered in some water bodies around the country^21,61,62,69^. The impacts of these and other medium to low-risk species on Malaysian freshwater ecosystems have probably not been extensively studied. Although most of the low-risk species from FIST (medium risk species from FISK) are known to be small-sized, guppy (*Poecilia reticulata*) which is also small-sized turned out to be of high-risk from both FIST and FISK. Furthermore, high-risk species like north African catfish (*Clarias gariepinus*) have been reported from native waters and recorded in aquaculture, a purpose for which it was introduced^6^.

Concerning high-risk species, both FIST and FISK gave similar outcomes by designating species such as north African catfish (*Clarias gariepinus*), carp (*Cyprinus carpio*), goldfish (*Carassius auratus*) and suckermouth catfish (*Hypostomus plecostomus*) as high-risk species. This shows that both tools are valuable, although, FISK v2 is likely more robust due to its higher number of questions, the inclusion of multiple elements that are considered to be valuable in predicting potential invasion success through previous invasion history, establishment success in other regions, climate matching and documented impacts^70,71^, and the ability to calibrate it for a specific risk assessment area^44,72,73^. The success of FISK v2 to discriminate between invasive and non-invasive fish species in this study is in line with earlier studies^14,44,72,73^. Besides, it has been suggested that before the application of FISK, a rapid screening tool may be utilized to flag the potentially high-risk species^73^. Therefore, FIST may be considered as a first-stage rapid risk screening tool^11^.

Despite being designated to be of high-risk, little or no reports regarding establishment in the wild exist for species such as carp (*Cyprinus carpio*)*,* goldfish (*Carassius auratus*) and oscar (*Astronotus ocellatus*) which also turned out to be of high risk. This is probably due to failed introduction owing to biotic and abiotic influences^44,74^, insufficient propagule pressure to guarantee the establishment of these species, or are yet to be detected in the wild. These species, therefore, appear to pose no considerable risk of invasiveness in the risk analysis area. This is similar to a report for some six high-risk species including goldfish and guppy in peninsular Florida, USA^44^.

All of the low-risk species from FIST turned out to be of medium risk according to FISK. Besides, some species known to have been established in local waters and of likely serious impact on biodiversity and environments like Amazon sailfin catfish (*Pterygoplichthys pardalis*)*,* vermiculated sailfin catfish (*Pterygoplichthys disjunctivus*) and peacock bass (*Cichla ocellaris*) are of medium risk according to FIST and of high-risk according to FISK. Again, this may be due to the robustness of FISK making it more accurate in designating the invasion status.

In this study, the calibrated high-risk threshold value of 17 is close to scores reported by previous studies ranging from 17 to 23, as reported by those that applied FISK v1^45,75,76^ and v2^12,13,46,77^. Contrarily, it is quite higher than the threshold values of 9.5, 11.75, and 10.5 from FISK v2 reported for the southern Balkan countries, northern Balkan countries (Croatia and Slovenia) and Florida, USA, respectively^10,44,72^. Moreover, the low range (3 to 7) of delta values and the negative relationship between the delta values and mean FISK scores indicate that the assessors generally have closer certainty levels for the species with higher scores than for the low scoring species. This differs from the wide range and positive relationship reported for the FISK v2 assessment of fish species from peninsular Florida, USA^44^. A possible reason for this is the higher number of assessors in their study which may have given rise to more divergent opinions.

This study represents the first attempt to risk-screen alien ornamental fish species in Peninsular Malaysia. We acknowledge the need for further studies to carry out screening of more species including those mainly for aquaculture which were not captured in this study. Moreover, there is a need for improved monitoring and management of the high-risk species where some of them are among the most popular ornamental fishes traded all over the world^3^. Priority should be given to those with established breeding populations, while those yet to establish should be prevented from getting introduced. In addition to the existing plan and efforts by the Malaysian government through the Department of Fisheries, Ministry of Agriculture and Food Industries, together with the Ministry of Energy and Natural Resources, aquarists and hobbyists should be involved as they are valuable in the successful monitoring and management fish introduction and invasion^3^. In case of the need to import new fish species in the future, an initial risk screening should be adopted to help hint on the possible impacts and guide policy decisions.

## Conclusion

Although noted as the second most important route for the introduction of alien fishes in Malaysia, the aquarium trade, judging from its composition of numerous alien fish species, could overtake aquaculture as a source of invasive fish introduction if these fishes continuously find their way into the local waters. The number of high-risk ornamental fish species gives an idea of a possible future invasion if unmonitored. Moreover, FISK is most likely a more robust tool in assessing the risk of invasion and for the most part, its outcome was commensurate with that of FIST with some differences. This also shows the value of FIST for an initial and rapid application.

## Supplementary information

Supplementary file1

Supplementary file2

Supplementary file3
