# Supplementary material for: Species composition and invasion risks of alien ornamental freshwater fishes from pet stores in Klang Valley, Malaysia
Source: Sci Rep. 2020 Oct 14;10:17205. doi: 10.1038/s41598-020-74168-9 (PMC7560888; doi:10.1038/s41598-020-74168-9)
Supplement: Supplementary file 1 — Supplementary file1 [file 41598_2020_74168_MOESM1_ESM.docx]

**Appendix 1** Checklist of alien freshwater fish species in the 60 ornamental fish pet stores within Klang Valley, Malaysia.

| **Family** | **Scientific name** | **Common name** | **Occurrence (%)** |
| --- | --- | --- | --- |
| Potamotrygonidae | *Potamotrygon leopoldi* Castex & Castello 1970 | White blotched stingray | 3.33 |
|  | *Potamotrygon motoro* (Müller & Henle 1841) | South American stingray | 6.67 |
| Polypteridae | *Polypterus ansorgii* Boulenger 1910 | Guinean bichir | 5.00 |
|  | *Polypterus delhezi* Boulenger 1899 | Barred bichir | 6.67 |
|  | *Polypterus bichir*Lacepède 1803 | Nile bichir | 8.33 |
|  | *Polypterus congicus* Boulenger 1898 | Congo bichir | 16.70 |
|  | *Polypterus endlicherii* Heckel 1847 | Saddled bichir | 21.70 |
|  | *Polypterus ornatipinnis* Boulenger 1902 | Ornate bichir | 16.70 |
|  | *Polypterus palmas* Ayres 1850 | Shortfin bichir | 8.33 |
|  | *Polypterus senegalus* Cuvier 1829 | Gray bichir | 30.00 |
| Acipenseridae | *Acipenser fulvescens* Rafinesque 1817 | Lake sturgeon | 8.33 |
| Polyodontidae | *Polyodon spathula* (Walbaum 1792) | Mississippi paddlefish | 1.67 |
| Lepisosteidae | *Lepisosteus oculatus* Winchell 1864 | Spotted gar | 5.00 |
|  | *Atractosteus spatula* (Lacepède 1803) | Alligator gar | 13.30 |
| Arapaimidae | *Arapaima gigas* (Schinz 1822) | Arapaima | 6.67 |
| Notopteridae | *Chitala blanci* (d'Aubenton 1965) | Royal knifefish | 5.00 |
|  | *Chitala ornata* (Gray 1831) | Clown knifefish | 18.30 |
| Mormyridae | *Campylomormyrus elephas* (Boulenger 1898) | Elephant nose fish | 3.33 |
| Gyrinocheilidae | *Gyrinocheilus aymonieri* (Tirant 1884) | Siamese algae-eater | 26.70 |
| Botiidae | *Botia lohachata* Chaudhuri 1912 | Reticulate loach | 1.67 |
|  | *Botia kubotai* Kottelat 2004 | Loach | 3.33 |
|  | *Botia striata* Narayan Rao 1920 | Zebra loach | 3.33 |
|  | *Chromobotia macracanthus* (Bleeker 1852) | Clown loach | 46.70 |
|  | *Syncrossus berdmorei*Blyth 1860 | Blyth's loach | 5.00 |
|  | *Syncrossus helodes* (Sauvage 1876) | Tiger botia | 1.67 |
|  | *Yasuhikotakia caudipunctata* (Taki & Doi 1995) | Laiksaba-takikala | 1.67 |
|  | *Yasuhikotakia lecontei* (Fowler 1937) | Silver loach | 1.67 |
|  | *Yasuhikotakia modesta*(Bleeker 1864) | Redtail botia | 6.67 |
| Cobitidae | *Pangio kuhlii* (Valenciennes 1846) | Coolie loach | 1.67 |
| Gastromyzontidae | *Sewellia lineolata* (Valenciennes 1846) | Sewilla | 5.00 |
| Cyprinidae | *Barbodes semifasciolatus* (Günther 1868) | Chinese barb | 1.67 |
|  | *Barbonymus altus* (Günther 1868) | Red tailed tinfoil | 48.30 |
|  | *Barbonymus gonionotus* Bleeker 1849 | Silver barb | 48.30 |
|  | *Carassius auratus* (Linnaeus 1758) | Goldfish | 83.30 |
|  | *Catlocarpio siamensis* Boulenger 1898 | Giant barb | 8.33 |
|  | *Crossocheilus reticulatus* (Fowler 1934) | Silver flying fox | 1.67 |
|  | *Cyprinus carpio* Linnaeus 1758 | Common carp | 73.30 |
|  | *Dawkinsia assimilis* (Jerdon 1849) | Mascara barb | 3.33 |
|  | *Epalzeorhynchos frenatum* (Fowler 1934) | Rainbow sharkminnow | 8.33 |
|  | *Garra flavatra* Kullander & Fang 2004 | Panda garra | 8.33 |
|  | *Haludaria fasciata* (Jerdon 1849) | Melon barb | 1.67 |
|  | *Neolissochilus hexagonolepis* (McClelland 1839) | NA | 5.00 |
|  | *Neolissochilus stracheyi* (Day 1871) | Thai's blue mahseer | 3.33 |
|  | *Neolissochilus sumatranus* (Weber & de Beaufort 1916) | Sumatra lauskmokk | 1.67 |
|  | *Pethia conchonius* (Hamilton 1822) | Rosy barb | 31.70 |
|  | *Pethia gelius* (Hamilton 1822) | Golden barb | 1.67 |
|  | *Pethia padamya* (Kullander & Britz 2008) | Odessa barb | 5.00 |
|  | *Puntioplites falcifer* Smith 1929 | Sicklefin barb | 1.67 |
|  | *Oliotius oligolepis* (Bleeker 1853) | Checkered barb | 1.67 |
|  | *Puntigrus tetrazona* (Bleeker 1855) | Sumatra barb | 76.67 |
|  | *Puntius titteya* Deraniyagala 1929 | Cherry barb | 13.30 |
|  | *Puntius viridis* Plamoottil & Abraham 2014 | Spot-fin green barb | 1.67 |
|  | *Rectoris posehensis* Lin 1935 | Rasbora | 3.33 |
|  | *Sahyadria denisonii* (Day 1865) | Denison's barb | 20.00 |
|  | *Tor putitora* (Hamilton 1822) | Putitor mahseer | 5.00 |
| Danionidae | *Rasbora daniconius* (Hamilton 1822) | Slender rasbora | 1.67 |
|  | *Danio rerio* (Hamilton 1822) | Zebrafish | 41.70 |
|  | *Rasbosoma spilocerca* (Rainboth & Kottelat 1987) | Dwarf scissorstail rasbora | 3.33 |
| Xenocyprididae | *Ctenopharyngodon idella* (Valenciennes 1844) | Grass carp | 1.67 |
|  | *Hypophthalmichthys molitrix* (Valenciennes 1844) | Silver carp | 1.67 |
| Tanichthyidae | *Tanichthys albonubes* Lin 1932 | White cloud mountain minnow | 1.67 |
| Distichodontidae | *Distichodus sexfasciatus* Boulenger 1897 | Sixbar distichodus | 6.67 |
| Alestidae | *Hydrocynus forskahlii* (Cuvier 1819) | Elongate tigerfish | 1.67 |
|  | *Hydrocynus goliath* Boulenger 1898 | Giant tiger fish | 3.33 |
|  | *Hydrocynus vittatus* Castelnau 1861 | Tigerfish | 5.00 |
| Hepsetidae | *Hepsetus odoe* (Bloch 1794) | African pike fish | 8.33 |
| Erythrinidae | *Hoplias aimara* (Valenciennes 1847) | Poisson tigre | 1.67 |
|  | *Hoplias malabaricus* (Bloch 1794) | Trahira | 1.67 |
| Cynodontidae | *Hydrolycus armatus* (Jardine 1841) | Payara | 1.67 |
| Serrasalmidae | *Metynnis hypsauchen* (Müller & Troschel 1844) | Silver dollar | 26.70 |
|  | *Piaractus brachypomus* (Cuvier 1818) | Pirapitinga | 5.00 |
|  | *Piaractus mesopotamicus* (Holmberg 1887) | Pacu | 3.33 |
|  | *Pygocentrus nattereri* Kner 1858 | Red piranha | 3.33 |
| Anostomidae | *Abramites hypselonotus* (Günther 1868) | Marbled headstander | 1.67 |
|  | *Leporinus steyermarki* Inger 1956 | Grauer leporinus | 1.67 |
|  | *Leporinus maculatus* Müller & Troschel 1844 | Spotted leporinus | 3.33 |
|  | *Leporinus fasciatus* (Bloch 1794) | Banded leporinus | 13.30 |
| Prochilodontidae | *Semaprochilodus insignis* (Jardine 1841) | Kissing prochilodus | 23.30 |
| Ctenoluciidae | *Ctenolucius hujeta* (Valenciennes 1850) | Gar charachin | 5.00 |
| Chalceidae | *Chalceus erythrurus* (Cope 1870) | Tucan fish | 5.00 |
| Gasteropelecidae | *Thoracocharax stellatus* (Kner 1858) | Spotfin hatchetfish | 1.67 |
| Bryconidae | *Salminus brasiliensis* (Cuvier 1816) | Dorado | 1.67 |
| Characidae | *Aphyocharax anisitsi* Eigenmann & Kennedy 1903 | Bloodfin tetra | 5.00 |
|  | *Gymnocorymbus ternetzi* (Boulenger 1895) | Black tetra | 41.70 |
|  | *Hasemania nana* (Lütken 1875) | Silvertip tetra | 16.70 |
|  | *Hemigrammus bleheri*Géry & Mahnert 1986 | Firehead tetra | 16.70 |
|  | *Hemigrammus erythrozonus* Durbin 1909 | Glowlight tetra | 1.67 |
|  | *Hyphessobrycon heterorhabdus* (Ulrey 1894) | Flag tetra | 1.67 |
|  | *Hyphessobrycon megalopterus* (Eigenmann 1915) | Black phantom tetra | 3.33 |
|  | *Hyphessobrycon amandae* Géry & Uj 1987 | Ember tetra | 1.67 |
|  | *Hyphessobrycon anisitsi* (Eigenmann 1907) | Buenos aires tetra | 15.00 |
|  | *Hyphessobrycon eques*(Steindachner 1882) | Jewel terta | 45.00 |
|  | *Hyphessobrycon herbertaxelrodi*Géry 1961 | Black neon tetra | 33.30 |
|  | *Hyphessobrycon pando* Hein 2009 | Pondo tetra | 1.67 |
|  | *Hyphessobrycon rosaceus* Durbin 1909 | Rosy tetra | 3.33 |
|  | *Moenkhausia sanctaefilomenae* (Steindachner 1907) | Redeye tetra | 33.30 |
|  | *Nematobrycon palmeri* Eigenmann 1911 | Emperor tetra | 1.67 |
|  | *Paracheirodon axelrodi* (Schultz 1956) | Cardinal tetra | 3.33 |
|  | *Paracheirodon innesi* (Myers 1936) | Neon tetra | 55.00 |
|  | *Pristella maxillaris* (Ulrey 1894) | X-ray tetra | 3.33 |
|  | *Thayeria boehlkei*Weitzman 1957 | Bllackline penguinfish | 18.30 |
| Apteronotidae | *Apteronotus albifrons* (Linnaeus 1766) | Black ghost | 21.70 |
| Callichthyidae | *Corydoras adolfoi* Burgess 1982 | Adolf's catfish | 1.67 |
|  | *Corydoras aeneus* (Gill 1858) | Bronze cory | 10.00 |
|  | *Corydoras agassizii* Steindachner 1876 | Agasizii | 1.67 |
|  | *Corydoras julii* Steindachner 1906 | Leopard corydoras | 1.67 |
|  | *Corydoras metae* Eigenmann 1914 | Masked corydoras | 1.67 |
|  | *Corydoras nattereri* Steindachner 1876 | Blue cory | 1.67 |
|  | *Corydoras paleatus* (Jenyns 1842) | Peppered cory | 21.70 |
|  | *Corydoras panda* Nijssen & Isbrücker 1971 | Panda cory | 5.00 |
|  | *Corydoras* *trilineatus* Cope 1872 | Three stripe corydoras | 6.67 |
|  | *Dianema urostriatum* (Miranda Ribeiro 1912) | Flagtail catfish | 1.67 |
| Loricariidae | *Ancistrus tamboensis* Fowler 1945 | Bristlenose catfish | 1.67 |
|  | *Ancistrus multispinis* (Regan 1912) | Barbadinho | 3.33 |
|  | *Ancistrus dolichopterus* Kner 1854 | Bushymouth catfish | 3.33 |
|  | *Hemiancistrus subviridis* Werneke, Sabaj Pérez, Lujan & Armbruster 2005 | Green phantom pleco | 3.33 |
|  | *Hypostomus plecostomus* (Linnaeus 1758) | Suckermouth catfish | 71.70 |
|  | *Hypostomus borellii* (Boulenger 1897) | Pleco | 3.33 |
|  | *Hypostomus alatus* Castelnau 1855 | Acarí | 1.67 |
|  | *Otocinclus vittatus* Regan 1904 | Limpa vidro | 6.67 |
|  | *Panaque nigrolineatus* (Peters 1877) | Royal panaque | 6.67 |
|  | *Pseudacanthicus pitanga* Chamon 2015 | Flame pleco | 3.33 |
|  | *Pterygoplichthys disjunctivus* (Weber 1991) | Vermiculated sailfin catfish | 8.33 |
|  | *Pterygoplichthys gibbiceps* (Kner 1854) | Leopard pleco | 15.00 |
|  | *Pterygoplichthys pardalis* (Castelnau 1855) | Amazon sailfin catfish | 13.30 |
| Pangasiidae | *Pangasianodon hypophthalmus* (Sauvage 1878) | Striped catfish | 41.70 |
| Siluridae | *Wallagonia micropogon* (Ng 2004) | Pa koon | 15.00 |
| Auchenipteridae | *Ageneiosus inermis* (Linnaeus 1766) | Bottlenose catfish | 1.67 |
|  | *Ageneiosus polystictus* Steindachner 1915 | Mandobé | 1.67 |
|  | *Trachelyopterus fisheri* (Eigenmann 1916) | Driftwood catfish | 1.67 |
|  | *Trachelyopterus striatulus* (Steindachner 1877) | Singing catfish | 1.67 |
|  | *Asterophysus batrachus* Kner 1858 | Bagre sapo | 3.33 |
| Doradidae | *Centrodoras hasemani* (Steindachner 1915) | NA | 1.67 |
|  | *Oxydoras sifontesi* Fernández-Yépez 1968 | Guitarrilla | 1.67 |
|  | *Platydoras armatulus* (Valenciennes 1840) | Southern striped raphel | 1.67 |
|  | *Platydoras costatus* (Linnaeus 1758) | Raphel catfish | 3.33 |
| Pimelodidae | *Brachyplatystoma tigrinum* (Britski 1981) | Tigerstriped catfish | 3.33 |
|  | *Brachyplatystoma juruense* (Boulenger 1898) | Zebra catfish | 1.67 |
|  | *Calophysus macropterus* (Lichtenstein 1819) | Zamurito | 1.67 |
|  | *Leiarius perruno* (Schultz 1944) | Leopard catfish | 1.67 |
|  | *Phractocephalus hemioliopterus* (Bloch & Schneider 1801) | Redtail catfish | 33.30 |
|  | *Pseudoplatystoma magdaleniatum* Buitrago-Suárez & Burr 2007 | Shovelnose tiger catfish | 1.67 |
| Clariidae | *Clarias gariepinus* (Burchell 1822) | North African catfish | 23.30 |
| Ariidae | *Neoarius graeffei* (Kner & Steindachner 1867) | Paupua shark catfish | 3.33 |
| Monodactylidae | *Monodactylus sebae* (Cuvier 1829) | African moony | 13.30 |
| Mochokidae | *Synodontis eupterus* Boulenger 1901 | Featherfin squeaker | 8.33 |
|  | *Synodontis multipunctatus* Boulenger 1898 | Dumi | 1.67 |
|  | *Synodontis nigriventris* David 1936 | Blotched upside-down catfish | 3.33 |
|  | *Synodontis notatus* Vaillant 1893 | Onespot squeaker | 11.70 |
| Claroteidae | *Auchenoglanis occidentalis* (Valenciennes 1840) | Bubu | 5.00 |
| Gobiidae | *Sicyopus jonklaasi* Klausewitz & Henrich 1986 | Lipstick goby | 3.33 |
| Anabantidae | *Ctenopoma acutirostre* Pellegrin 1899 | Spotted ctenopoma | 3.33 |
| Channidae | *Channa asiatica* (Linnaeus 1758) | Small snakehead | 1.67 |
| Cichlidae | *Amatitlania nigrofasciata* (Günther 1867) | Convict cichlid | 5.00 |
|  | *Andinoacara latifrons* (Steindachner 1878) | Platinum acara | 1.67 |
|  | *Andinoacara rivulatus* (Günther 1860) | Green terror | 5.00 |
|  | *Astronotus crassipinnis* (Heckel 1840) | Apaiari | 3.33 |
|  | *Astronotus ocellatus* (Agassiz 1831) | Oscar | 61.70 |
|  | *Aulonocara nyassae* Regan 1922 | Emperor cichlid | 3.33 |
|  | *Aulonocara jacobfreibergi*(Johnson 1974) | Fairy cichlid | 6.67 |
|  | *Biotodoma cupido* (Heckel 1840) | Green streaked eartheater | 1.67 |
|  | *Chindongo demasoni*(Konings 1994) | Demasons cichlid | 8.33 |
|  | *Chindongo elongatus*(Fryer 1956) | Elongate mbuna | 1.67 |
|  | *Chindongo socolofi* (Johnson 1974) | Pindani | 3.33 |
|  | *Cichla* spp. | Parrot fish | 70.00 |
|  | *Cichla intermedia* Machado-Allison 1971 | Pavon real | 1.67 |
|  | *Cichla melaniae* Kullander & Ferreira 2006 | NA | 3.33 |
|  | *Cichla monoculus*Spix & Agassiz 1831 | Tucanare peacock bass | 6.67 |
|  | *Cichla ocellaris*Bloch & Schneider 1801 | Peacock bass | 11.70 |
|  | *Cichla orinocensis*Humboldt 1821 | Orinoco Peacock bass | 8.33 |
|  | *Cichla pinima* Kullander & Ferreira 2006 | Pinima bass | 3.33 |
|  | *Cichla piquiti* Kullander & Ferreira 2006 | Azul peacock | 10.00 |
|  | *Cichla temensis*Humboldt 1821 | Speckled pavon | 8.33 |
|  | *Amphilophus trimaculatus* (Günther 1867) | Threespot cichlid | 1.67 |
|  | Cichlid hybrid | Flowerhorn | 61.70 |
|  | *Copadichromis borleyi* (Iles 1960) | Haplochromis borleyi redfin | 1.67 |
|  | *Crenicichla alta* Eigenmann 1912 | Millet pike | 1.67 |
|  | *Crenicichla lugubris* Heckel 1840 | Red pike cichlid | 5.00 |
|  | *Cyphotilapia frontosa* (Boulenger 1906) | Humphead cichlid | 11.70 |
|  | *Dimidiochromis compressiceps* (Boulenger 1908) | Malawi eyebiter cichlid | 1.67 |
|  | *Dimidiochromis dimidiatus* (Günther 1864) | Ncheni type haplochromis | 1.67 |
|  | *Geophagus surinamensis* (Bloch 1791) | Red striped eartheater | 21.70 |
|  | *Geophagus altifrons*Heckel 1840 | Altifrons eartheater | 8.33 |
|  | *Geophagus brachybranchus* Kullander & Nijssen 1989 | Mangotonkija | 1.67 |
|  | *Geophagus dicrozoster* López-Fernández & Taphorn 2004 | Dicrozoster eartheater | 3.33 |
|  | *Geophagus steindachneri* Eigenmann & Hildebrand 1922 | Redhump eartheater | 1.67 |
|  | *Guianacara geayi* (Pellegrin 1902) | Krobia | 3.33 |
|  | *Guianacara dacrya* Arbour & López-Fernández 2011 | NA | 1.67 |
|  | *Guianacara sphenozona* Kullander & Nijssen 1989 | Ouru muje | 1.67 |
|  | *Gymnogeophagus balzanii* (Perugia 1891) | Paraguay mouthbrooder | 3.33 |
|  | *Hemichromis bimaculatus*Gill 1862 | Jewelfish | 25.00 |
|  | *Herichthys cyanoguttatus* Baird & Girard 1854 | Rio grande cichlid | 3.33 |
|  | *Heterotilapia buttikoferi* (Hubrecht 1881) | Zebra tilapia | 5.00 |
|  | *Labeotropheus fuelleborni* Ahl 1926 | Blue mbuna | 1.67 |
|  | *Labeotropheus trewavasae* Fryer 1956 | Scrapemouth mbuna | 1.67 |
|  | *Labidochromis caeruleus*Fryer 1956 | Blue streak hap | 8.33 |
|  | *Maskaheros argenteus* (Allgayer 1991) | White cichlid | 1.67 |
|  | *Mayaheros urophthalmus* (Günther 1862) | Mexican mojarra | 3.33 |
|  | *Melanochromis auratus* (Boulenger 1897) | Golden mbuna | 5.00 |
|  | *Mesoheros festae* (Boulenger 1899) | Guayas cichlis | 1.67 |
|  | *Mesonauta festivus* (Heckel 1840) | Flag cichlid | 1.67 |
|  | *Mikrogeophagus ramirezi* (Myers & Harry 1948) | Ram cichlid | 35.00 |
|  | *Neolamprologus leleupi* (Poll 1956) | Lemon cichlid | 18.30 |
|  | *Nimbochromis livingstonii* (Günther 1894) | Livingstone-cichlide | 13.30 |
|  | *Nimbochromis venustus* (Boulenger 1908) | Venustus | 5.00 |
|  | *Oreochromis* hybrid | Red tilapia | 11.70 |
|  | *Parachromis friedrichsthalii* (Heckel 1840) | Guapote armarillo | 3.33 |
|  | *Paretroplus menarambo* Allgayer 1996 | Menarambo cichlid | 1.67 |
|  | *Placidochromis milomo* Oliver 1989 | Super VC-10 hap | 5.00 |
|  | *Pseudotropheus cyaneorhabdos* (Bowers & Stauffer 1997) | Maingano | 3.33 |
|  | *Pseudotropheus johannii*Eccles 1973 | Bluegray mbuna | 6.67 |
|  | *Pterophyllum scalare* (Schultze 1823) | Freshwater angelfish | 81.70 |
|  | *Satanoperca daemon* (Heckel 1840) | Threespot eartheather | 1.67 |
|  | *Sciaenochromis ahli* (Trewavas 1935) | Electric blue hap | 6.67 |
|  | *Symphysodon aequifasciatus* Pellegrin 1904 | Blue discuss | 31.70 |
|  | *Symphysodon tarzoo* Lyons 1959 | Discuss | 1.67 |
|  | *Thorichthys maculipinnis* (Steindachner 1864) | Spotcheek cichlid | 1.67 |
|  | *Trichromis salvini* (Günther 1862) | Yellow belly cichlid | 3.33 |
|  | *Tropheus duboisi*Marlier 1959 | Spotted dubosi | 5.00 |
|  | *Uaru amphiacanthoides*Heckel 1840 | Uaru | 5.00 |
|  | *Uaru fernandezyepezi* Stawikowski 1989 | Uaru | 1.67 |
|  | *Vieja maculicauda* (Regan 1905) | Blackbelt cichlid | 1.67 |
|  | *Vieja melanurus* (Günther 1862) | Redhead cichlid | 5.00 |
|  | *Vieja zonata* (Meek 1905) | Oaxaca cichlid | 1.67 |
| Melanotaeniidae | *Glossolepis incisa* Weber 1907 | Red rainbowfish | 8.33 |
|  | *Iriatherina werneri* Meinken 1974 | Threadfin rainbow | 1.67 |
|  | *Melanotaenia praecox* (Weber de & Beaufort 1922) | Dwarf rainbow | 6.67 |
|  | *Melanotaenia boesemani* Allen & Cross 1980 | Boseman rainbowfish | 11.70 |
|  | *Melanotaenia caerulea* Allen 1996 | Blue rainbowfish | 3.33 |
|  | *Melanotaenia parkinsoni* Allen 1980 | Parkinson's rainbowfish | 1.67 |
| Poeciliidae | *Poecilia latipinna* (Lesueur 1821) | Molly | 56.70 |
|  | *Poecilia wingei* Poeser Kempkes & Isbrücker 2005 | Guppy | 6.67 |
|  | *Poecilia reticulata* Peters 1859 | Guppy | 66.70 |
|  | *Poecilia sphenops* Valenciennes 1846 | Molly | 18.30 |
|  | *Xiphophorus hellerii* Heckel 1848 | Green swordtail | 15.00 |
|  | *Xiphophorus maculatus* (Günther 1866) | Southern platyfish | 15.00 |
| Procatopodidae | *Poropanchax normani* (Ahl 1928) | Norman's lampeye | 1.67 |
| Osphronemidae | *Betta splendens* Regan 1910 | Siamese fighting fish | 38.30 |
|  | *Trichopodus microlepis* (Günther 1861) | Moonlight gourami | 11.70 |
|  | *Macropodus opercularis* (Linnaeus 1758) | Paradisefish | 13.30 |
|  | *Trichopodus pectoralis* Regan 1910 | Snakeskin gourami | 21.70 |
|  | *Trichogaster lalius* (Hamilton 1822) | Dwarf gourami | 36.70 |
| Lobotidae | *Datnioides pulcher* (Kottelat 1998) | Siamese tiger perch | 13.30 |
|  | *Datnioides undecimradiatus* (Roberts & Kottelat 1994) | Mekong tiger perch | 15.00 |
| Tetraodontidae | *Carinotetraodon travancoricus* (Hora & Nair 1941) | Malabar pufferfish | 1.67 |
|  | *Dichotomyctere nigroviridis* (Marion de Procé 1822) | Spotted green pufferfish | 3.33 |
|  | *Tetraodon lineatus* Linnaeus 1758 | Globe fish | 3.33 |
|  | *Tetraodon mbu* Boulenger 1899 | Fresh water puffer fish | 1.67 |
|  | *Tetraodon miurus* Boulenger 1902 | Mbubo | 1.67 |
|  | *Tetraodon schoutedeni* Pellegrin 1926 | Giant puffer | 1.67 |
| Centrarchidae | *Lepomis auritus* (Linnaeus 1758) | Red breast sunfish | 5.00 |

NA: Not available
