# Supplementary material for: Species composition and invasion risks of alien ornamental freshwater fishes from pet stores in Klang Valley, Malaysia
Source: Sci Rep. 2020 Oct 14;10:17205. doi: 10.1038/s41598-020-74168-9 (PMC7560888; doi:10.1038/s41598-020-74168-9)
Supplement: Supplementary file 2 — Supplementary file2 [file 41598_2020_74168_MOESM2_ESM.docx]

**Appendix 2** Checklist of native freshwater fish species in the 60 ornamental fish pet stores within Klang Valley, Malaysia.

| **Family** | **Scientific name** | **Common name** |
| --- | --- | --- |
| Osteoglossidae | *Scleropages formosus* (Müller & Schlegel 1840) | Arowana |
| Notopteridae | *Notopterus notopterus* (Pallas 1769) | Bronze featherback |
| Cyprinidae | *Epalzeorhynchos kalopterum* (Bleeker 1850) | Flying fox |
|  | *Osteochilus vittatus* (Valenciennes 1842) | Bonylip barb |
|  | *Osteochilus melanopleura* (Bleeker 1852) | Greater bony lipped barb |
|  | *Puntioplites bulu* (Bleeker 1851) | Crossbanded barb |
|  | *Hypsibarbus wetmorei* (Smith 1931) | Golden belly barb |
|  | *Neolissochilus soroides* (Duncker 1904) | Copper mahseer |
|  | *Tor tambra* (Valenciennes 1842) | Malayan mahseer |
|  | *Barbodes lateristriga* (Valenciennes 1842) | Spanner barb |
|  | *Crossocheilus oblongus* Kuhl & van Hasselt 1823 | Siamese flying fox |
|  | *Probarbus jullieni* Sauvage 1880 | Jullien's golden carp |
|  | *Systomus rubripinnis* (Valenciennes 1842) | Javaen barb |
|  | *Labeo chrysophekadion* (Bleeker 1849) | Black sharkminnow |
|  | *Hampala macrolepidota* Kuhl & van Hasselt 1823 | Hampala barb |
|  | *Balantiocheilos melanopterus* (Bleeker 1850) | Tricolor sharkminnow |
|  | *Barbonymus schwanefeldii* (Bleeker 1854) | Tinfoil barb |
| Danionidae | *Rasbora trilineata* Steindachner 1870 | Three-lined rasbora |
|  | *Trigonostigma heteromorpha* (Duncker 1904) | Harlequin rasbora |
| Leptobarbidae | *Leptobarbus hoevenii* (Bleeker 1851) | Hoven’s carp |
| Latidae | *Lates calcarifer* (Bloch 1790) | Freshwater Asian seabass |
| Bagridae | *Pseudomystus leiacanthus* (Weber & de Beaufort 1912) | Dwarf bumble bee catfish |
| Siluridae | *Phalacronotus apogon* (Bleeker 1851) | Segahak Kalimantan |
|  | *Kryptopterus bicirrhis* (Valenciennes 1840) | Glass catfish |
| Clariidae | *Clarias batrachus* (Linnaeus 1758) | Catfish |
| Toxotidae | *Toxotes chatareus* (Hamilton 1822) | Spotted archerfish |
|  | *Toxotes jaculatrix* (Pallas 1767) | Banded archerfish |
| Eleotridae | *Oxyeleotris marmorata* (Bleeker 1852) | Marble goby |
| Mastacembelidae | *Mastacembelus erythrotaenia* Bleeker 1850 | Fire eel |
| Gobiidae | *Brachygobius doriae* (Günther 1868) | Bumblebee goby |
| Anabantidae | *Anabas testudineus* (Bloch 1792) | Climbing perch |
| Helostomatidae | *Helostoma temminckii* Cuvier 1829 | Kissing gourami |
| Channidae | *Channa striata* (Bloch 1793) | Snakehead |
|  | *Channa marulioides* (Bleeker 1851) | Emperor snakehead |
|  | *Channa micropeltes* (Cuvier 1831) | Giant snakehead |
| Osphronemidae | *Betta bellica* Sauvage 1884 | Slim betta |
|  | *Trichopodus leerii* (Bleeker 1852) | Pearl gourami |
|  | *Osphronemus goramy* Lacepède 1801 | Giant gourami |
|  | *Trichopodus trichopterus* (Pallas 1770) | Three spot gourami |
| Lobotidae | *Datnioides microlepis* Bleeker 1854 | Finescale tigerfish |
| Scatophagidae | *Scatophagus argus* (Linnaeus 1766) | Spotted scat |
| Tetraodontidae | *Dichotomyctere ocellatus* (Steindachner 1870) | Eyespot pufferfish |
