# Supplementary material for: Species composition and invasion risks of alien ornamental freshwater fishes from pet stores in Klang Valley, Malaysia
Source: Sci Rep. 2020 Oct 14;10:17205. doi: 10.1038/s41598-020-74168-9 (PMC7560888; doi:10.1038/s41598-020-74168-9)
Supplement: Supplementary file 3 — Supplementary file3 [file 41598_2020_74168_MOESM3_ESM.docx]

Article

Species composition and invasion risks of alien ornamental freshwater fishes from pet stores in Klang Valley, Malaysia

Abdulwakil Olawale Saba^1,2^, Ahmad Ismail^1^, Syaizwan Zahmir Zulkifli^1^, Muhammad Rasul Abdullah Halim^3^, Noor Azrizal Abdul Wahid^4^ & Mohammad Noor Azmai Amal^1,*^

^1^Department of Biology, Faculty of Science, Universiti Putra Malaysia, 43400 UPM Serdang, Selangor, Malaysia

^2^School of Agriculture, Lagos State University, Epe Campus, 106101, Epe, Lagos, Nigeria

^3^School of Biological Sciences, Universiti Sains Malaysia, 11800 Gelugor, Penang, Malaysia

^4^Institute of Advanced Studies, University of Malaya, 50603 Kuala Lumpur, Malaysia

*^*^*e-mail: mnamal@upm.edu.my
